# Age-specific effects of screen time on overweight/obese: a structural equation model of children and adolescents in Western Maharashtra, India

**DOI:** 10.3389/fpubh.2025.1682181

**Published:** 2026-01-02

**Authors:** Shraddha Thorat, Rupeshkumar Deshmukh, Saibal Adhya

**Affiliations:** Department of Community Medicine, Bharati Vidyapeeth (Deemed to be University) Medical College, Pune, India

**Keywords:** age-specific, overweight/obese, children, adolescent, screen time

## Abstract

**Background:**

The rapid increase in digital technology use has led to unprecedented screen time levels among children and adolescents, raising concerns about overweight/obesity, especially in urban settings. Despite growing global evidence, limited data exists for Indian cities like Pune.

**Objectives:**

This cross-sectional study assessed the relationship between screen time duration and overweight/obesity in children’s (6–11 years) and adolescents (12–19 years) in Pune, India, accounting for dietary patterns, physical activity, and sociodemographic factors.

**Methods:**

A sample of 3,920 students from urban Pune schools was evaluated for screen time, physical activity, diet, BMI, and sociodemographic variables. Multinomial logistic regression and structural equation modeling identified associations between these factors and overweight/obesity, stratified by age groups (6–11 and 12–19 years).

**Results:**

Overweight/obesity prevalence was 27.1%. Excessive screen time was strongly associated with higher overweight and obesity risk. Children with 2–4 h daily screen time had 35.69 times higher Adj. OR (*p* < 0.001), and those exceeding 4 h had 28.80 times higher Adj. OR (*p* < 0.001). Among children’s (6–11 years), 2–4 h increased Adj. OR by 88.58 times (*p* < 0.001) and over 4 h by 69.45 times (*p* < 0.001). In adolescents (12–19 years), 2–4 h increased Adj. OR by 11.88 times (*p* < 0.001) and over 4 h by 9.22 times (*p* < 0.001).

**Conclusion:**

High screen time is a major modifiable risk factor for obesity among urban children and adolescents in Pune. Age-specific and family-centered interventions targeting reduced screen time and improved physical activity are urgently needed for effective obesity prevention and health promotion.

## Introduction

The growing integration of digital technology into everyday life has significantly changed lifestyle patterns, especially among children and adolescents. Screen time—which includes activities such as watching television, playing video games, and using smartphones or computers—has surged to unprecedented levels globally. This increase in sedentary behavior raises substantial public health concerns, particularly when viewed alongside the rising prevalence of obesity in children and adolescents. Obesity in this age group is a complex public health issue linked to various negative health outcomes that can persist into adulthood, including metabolic disorders, cardiovascular diseases, and psychological distress. Higher levels of physical activity and outdoor play marked Childhood (1). However, since the mid-20th century, with the introduction of televisions, followed by personal computers, gaming consoles, and more recently, mobile devices and the internet, children’s leisure activities have undergone a significant transformation. This technological shift has gradually reduced opportunities for active play, replacing them with screen-based entertainment, and, more recently, online learning and social interaction (2). The COVID-19 pandemic further accelerated this trend, as lockdowns resulted in a substantial increase in screen time for both educational and recreational purposes.

The issue of childhood and adolescent obesity is a significant global health challenge. As of 2022, more than 390 million children and adolescents aged 5 to 19 years were classified as overweight, including 160 million who were living with obesity. The prevalence of overweight (including obesity) in this age group has skyrocketed from just 8% in 1990 to 20% in 2022. In India, childhood obesity is also emerging as a serious public health concern. A pooled estimate indicates a prevalence of 9.0% in urban areas, compared to 4.0% in rural areas. Recent prevalence data specifically linking screen time to obesity among children aged 6–19 in Pune is limited, but existing studies present a concerning overview (3). One study conducted from 2009 to 2011 among school children aged 10–15 years reported an overall prevalence of overweight at 9.99% and obesity at 5.62%. More recently, a study of secondary school children in rural Pune revealed a high prevalence of excessive screen time, with 83.2% of students exceeding the recommended limits, a situation largely influenced by the pandemic (4). While these figures indicate significant individual issues, a direct and current link between increased screen time and obesity among the specified age group in urban Pune has not been thoroughly explored.

Globally, an increasing evidence links excessive screen time with higher body fat and unhealthy eating behaviors. However, a substantial research gap persists in understanding the relationship between total screen time duration and obesity among children and adolescents aged 6 to 19 years in urban Indian settings. Existing studies from India have largely focused on rural populations or specific subgroups, offering limited insights into how prolonged screen exposure correlates with obesity indicators such as Body Mass Index (BMI) and waist circumference in metropolitan areas like Pune.

The present study aimed to address this gap by estimating the prevalence of excessive screen time and obesity among school-going children and adolescents in Pune and by examining the association between screen time duration and obesity. The study also considered the potential influence of physical activity and dietary habits on this relationship. Conducted as a cross-sectional study across urban schools, it provides age-specific insights that enhance the understanding of digital behavior and obesity risk in this demographic.

The findings are expected to inform evidence-based public health strategies that promote balanced screen use, active lifestyles, and healthy dietary behaviors among children and adolescents in urban India. By focusing on a rapidly urbanizing region, this study adds valuable evidence to the growing global discourse on the health impacts of modern digital lifestyles.

Primary Objective of the study was to assess the association between daily screen time and the risk of overweight and obesity among children and adolescents aged 6–19 years. Secondary Objectives were to evaluate the dose–response relationship between screen time duration and BMI categories and to examine socio-demographic correlates (age, gender, socioeconomic status) influencing screen time, physical activity with overweight/obesity.

## Methodology

### Study design

This study was a cross-sectional study conducted in urban schools located in Pune, Maharashtra, India.

### Study setting

The study was carried out in urban schools of Pune city in Maharashtra state, India. The study population consisted of children and adolescents enrolled in grades 1 to 12 (aged 6–19 years) in selected urban schools in Pune. Inclusion Criteria were students aged 6–19 years enrolled in grades 1 to 12 in schools. Students whose parents or guardians provided informed consent and students who provided assent (for those aged more and equal to 10 years). Exclusion Criteria were students with diagnosed medical conditions affecting body weight (e.g., endocrine disorders, genetic syndromes). Students with physical disabilities limit physical activity and students absent during the data collection period.

Ethical approval was obtained from the Institutional Ethics Committee prior to the commencement of study. Informed consent was secured from parents or guardians of all participating students, ensuring they were fully informed about the study’s purpose, procedures, potential risks, and benefits. For students aged 10 years and older, assent was also obtained to respect their autonomy and willingness to participate.

### Sample size

The sample size calculation was done by n-master software using the formula *n* = (Z₁_−_*α*/₂)^2^ × p × q × (1 + (m−1)*ρ*)/d^2^, based on cluster design for estimating the prevalence of overweight/obese (30.7%), as reported in a published study by Bakour et al. (5), assuming a school as a cluster, an intra-cluster correlation (ICC) of 0.11, and a cluster size of 100 children per school, the required sample size was determined. With an absolute difference (d) of 5% and a 95% confidence level, the calculated minimum sample size for this study is 3,887 school children in Pune city.

### Sampling method

A multistage cluster random sampling method was employed to achieve the sample size of 3,920 participants, ensuring representativeness across socioeconomic and demographic groups in urban Pune. In the Stage 1, selection of schools was done obtaining comprehensive list of all urban schools in Pune was compiled, categorized by type (public and private) to reflect socioeconomic diversity. Total 1,254 schools are there in Pune district. Out of that 177 schools met eligibility criteria. This included being located in urban areas, offering grades 1 to 12, having a minimum enrollment of 100 students, and obtaining permission from school authorities for participation. Schools were selected based on geographic zones (e.g., central, eastern, western Pune) to ensure coverage across the city. Forty schools were randomly selected using a random number generator to achieve a balanced representation. At stage 2 selection of classes such as within each selected school, a list of classes from grades 1 to 12 was obtained. Four to six classes per school were randomly selected, proportional to the school’s enrollment size, ensuring representation across primary (grades 1–6) and upper primary and secondary (grades 7–12) levels. The number of classes selected was adjusted to meet the target sample size per school (approximately 100 students per school: 3,920 ÷ 40 schools). At the stage 3 selection of students were from each selected class, 20–25 students were randomly selected using class rosters and a random number generator. If a selected student was ineligible (based on inclusion/exclusion criteria) or declined participation, they were replaced with another randomly selected student from the same class. This multistage approach ensured a representative sample while accounting for clustering effects within schools and classes.

### Research tool

A pretested and pre-validated structured questionnaire was used to collect data via face-to-face interviews with students. The questionnaire covered Socio-demographic details (age, gender, socioeconomic status using the Modified Kuppuswamy Scale May 2025), Daily screen time (hours spent on devices such as smartphones, tablets, computers, and televisions) and Physical activity levels (frequency and duration of moderate-to-vigorous activities). Validity of the questionnaire was pretested on 30 students (not included in the final sample) to ensure clarity and reliability.

### Data collection

Data were collected by two trained field supervisors (FS) under the supervision of the research team. Field supervisors (FS) underwent a 2-day training session on administering the questionnaire, conducting anthropometric measurements. Training included mock interviews and standardization of measurement techniques. Trained field supervisors (FS) measured height (using a stadiometer) and weight (using a calibrated digital scale) to calculate Body Mass Index (BMI). Measurements followed WHO guidelines for standardization.

### Statistical analysis

All statistical analyses were performed using SPSS software (version 29) and R-software with version 4.5.1. Quantitative variables were presented as mean ± standard deviation (SD), while qualitative variables were expressed as frequencies and percentages (%), with graphical representations included where appropriate.

Initially, chi-square tests were performed to examine bivariate associations between categorical variables and BMI categories, assessing the statistical significance of relationships before proceeding to multivariable modeling. Multinomial logistic regression to examine factors associated with BMI categories among 3,920 children and adolescents aged 6–19 years, with Normal BMI serving as the reference category against Underweight and Overweight/Obese classifications. The analysis incorporated demographic variables (gender, religion, age group), parental socioeconomic factors (father/mother education and occupation), and behavioral determinants (diet type, physical activity, screen time) as independent predictors. Multivariate multinomial logistic regression analysis was specifically applied for the main exposure variable screen time after adjusting for all variables, to determine the independent association between screen time exposure and BMI categories while controlling for potential confounders. Odds ratios with 95% confidence intervals were calculated to quantify associations, with model fit assessed using likelihood ratio tests and pseudo R-squared values. Additionally, Structural Equation Modeling (SEM) was employed to examine complex interrelationships between latent constructs and BMI Overweight/Obese outcomes, particularly focusing on pathways leading to Overweight/Obese categories, with model adequacy evaluated using standard fit indices (CFI > 0.95, TLI > 0.95, RMSEA <0.06, SRMR <0.08). Age-stratified analyses were performed to identify developmental differences between younger (6–11 years) and older (12–19 years) cohorts, with interaction effects tested to determine if associations varied by age group. All analyses were conducted using maximum likelihood estimation with statistical significance set at *α* = 0.05, and both direct and indirect pathways were examined through the SEM framework to understand the mediating role of behavioral factors in the relationship between sociodemographic characteristics and BMI outcomes.

### Definitions used in the present study

1. Screen time: the total number of hours per day spent using electronic devices (e.g., smartphones, tablets, computers, televisions) for recreational purposes, as reported by the student or parent.
2. Overweight and obesity: overweight – BMI greater than or equal to 25 to 29.9 kg/m^2, obesity – BMI greater than or equal to 30 kg/m^2.
3. Physical activity: moderate-to-vigorous physical activity (MVPA) lasting at least 45 min per day, as recommended by ICMR, measured via self-reported frequency and duration.

## Results

In the total data, the gender distribution was 52.0% male and 48.0% female, with the majority being Hindu (81.6%) and following a mixed diet (74.5%). Among 6–11 years children (*n* = 1,261), the gender distribution was nearly equal (50.4% female, 49.6% male), with 65.9% Hindu and 20.1% Muslim representation. Physical activity was reported by 60.6% of children, and screen time was predominantly <1 h (36.2%) and 1–2 h (37.4%). The BMI distribution showed 63.6% normal weight, 26.1% overweight/obese, and 10.3% underweight. Among 12–19 years adolescents (*n* = 2,659), males comprised 53.2% and females 46.8%, with 89.1% Hindu and 5.8% Muslim. Physical activity was reported by 60.0% of adolescents, with screen time primarily 1–2 h (58.4%) or 2–4 h (29.2%). The BMI distribution revealed 58.8% normal weight, 27.6% overweight/obese, and 13.6% underweight. The overweight/obese prevalence was 27.1% in the total data, 26.1% among children, and 27.6% among adolescents (Table 1).

**Table 1:** Frequency distribution of sociodemographic, behavioral, and anthropometric variables among study participants (*n* = 3,920).

Demographic characteristics/risk factors		Total (*n* = 3,920)		6–11 years children’s (*n* = 1,261)		12–19 years adolescent (*n* = 2,659)	
		Frequency (*n*)	Percentage (%)	Frequency (*n*)	Percentage (%)	Frequency (*n*)	Percentage (%)
Gender	Female	1,881	48.0	636	50.4	1,245	46.8
	Male	2,039	52.0	625	49.6	1,414	53.2
Religion	Muslim	408	10.4	253	20.1	155	5.8
	Buddhist	163	4.2	94	7.5	69	2.6
	Hindu	3,200	81.6	831	65.9	2,369	89.1
	Other	149	3.8	83	6.6	66	2.5
Diet	Veg	998	25.5	368	29.2	630	23.7
	Mixed	2,922	74.5	893	70.8	2029	76.3
Father education	Illiterate	130	3.3	42	3.3	88	3.3
	Read and write	280	7.1	106	8.4	174	6.5
	Primary (1–4)	400	10.2	125	9.9	275	10.3
	Middle (5–7)	630	16.1	200	15.9	430	16.2
	Secondary (8–10)	1,800	45.9	576	45.7	1,224	46.0
	Higher secondary (11–12)	500	12.8	157	12.5	343	12.9
	Diploma after 10th	20	0.5	6	0.5	14	0.5
	Graduate	160	4.1	49	3.9	111	4.2
Mother education	Illiterate	100	2.6	37	2.9	63	2.4
	Primary (1–4)	1,000	25.5	326	25.9	674	25.3
	Middle (5–7)	960	24.5	308	24.4	652	24.5
	Secondary (8–10)	1,430	36.5	457	36.2	973	36.6
	Higher secondary (11–12)	380	9.7	118	9.4	262	9.9
	Graduate	50	1.3	15	1.2	35	1.3
Father occupation	Business	100	2.6	32	2.5	68	2.6
	Service	420	10.7	136	10.8	284	10.7
	Skilled labor	1,580	40.3	499	39.6	1,081	40.7
	Unskilled labor	1820	46.4	594	47.1	1,226	46.1
Mother occupation	Business	20	0.5	6	0.5	14	0.5
	Service	350	8.9	117	9.3	233	8.8
	Skilled labor	470	12.0	146	11.6	324	12.2
	Unskilled labor	1,210	30.9	397	31.5	813	30.6
	Housewife	1870	47.7	595	47.2	1,275	48.0
Physical activity	No	1,560	39.8	497	39.4	1,063	40.0
	Yes	2,360	60.2	764	60.6	1,596	60.0
Screen time in hours	1–2 h	2024	51.6	471	37.4	1,553	58.4
	2–4 h	1,047	26.7	271	21.5	776	29.2
	>4 h	190	4.8	62	4.9	128	4.8
	<1 h	659	16.8	457	36.2	202	7.6
BMI category	Normal	2,366	60.4	802	63.6	1,564	58.8
	Overweight/obese	1,063	27.1	329	26.1	734	27.6
	Underweight	491	12.5	130	10.3	361	13.6

Association between BMI status and various factors across the total and age-specific groups. Screen time demonstrated the strongest association with BMI in all groups (total: χ^2^ = 1727.30, *p* < 0.001; children: χ^2^ = 570.78, *p* < 0.001; adolescents: χ^2^ = 1336.84, *p* < 0.001), indicating this is the most influential lifestyle factor. Physical activity also showed consistent significant associations across total (χ^2^ = 130.46, *p* < 0.001), children (χ^2^ = 75.77, *p* < 0.001), and adolescents (χ^2^ = 62.74, *p* < 0.001). Religion maintained significant associations in all groups (total: χ^2^ = 133.80; children: χ^2^ = 38.39; adolescents: χ^2^ = 165.22; all *p* < 0.001). Notably, gender showed significant association in the total sample (χ^2^ = 54.06, *p* < 0.001) and adolescents (χ^2^ = 109.33, *p* < 0.001) but not in children (χ^2^ = 4.76, *p* = 0.09), suggesting gender-based BMI differences emerge during adolescence. Similarly, diet type was significantly associated with BMI in the total sample (χ^2^ = 48.76, *p* < 0.001) and adolescents (χ^2^ = 83.90, *p* < 0.001) but not in children (χ^2^ = 1.13, *p* = 0.57). Father’s education showed the opposite pattern, being significant in children (χ^2^ = 8.07, *p* = 0.02) but not adolescents (χ^2^ = 2.66, *p* = 0.26). Mother’s occupation was associated with BMI in children (χ^2^ = 13.09, *p* = 0.04) but not adolescents (χ^2^ = 9.57, *p* = 0.15), while mother’s education and father’s occupation showed no significant associations in any group (Table 2).

**Table 2:** Associations between sociodemographic, behavioral factors and risk factors with BMI categories.

Demographic characteristics/risk factors		Total						6–11 years						12–19 years					
		BMI			Total	Chi-square value	*p*-value	BMI			Total	Chi-square value	*p*-value	BMI			Total	Chi-square value	*p*-value
		Overweight/obese	Underweight	Normal				Overweight/obese	Underweight	Normal				Overweight/obese	Underweight	Normal			
Gender	Female	583	275	1,023	1881	54.06	**<0.001**	154	59	423	636	4.76	0.09	429	216	600	1,245	109.33	**<0.001**
	Male	480	216	1,343	2039			175	71	379	625			305	145	964	1,414		
Religion	Muslim	112	31	265	408	133.80	**<0.001**	58	9	186	253	38.39	<0.001	54	22	79	155	165.22	**<0.001**
	Buddhist	23	49	91	163			15	17	62	94			8	32	29	69		
	Hindu	875	363	1962	3,200			226	89	516	831			649	274	1,446	2,369		
	Other	53	48	48	149			30	15	38	83			23	33	10	66		
Diet	Veg	350	90	558	998	48.76	**<0.001**	89	37	242	368	1.13	0.57	261	53	316	630	83.90	**<0.001**
	Mixed	713	401	1,808	2,922			240	93	560	893			473	308	1,248	2029		
Father education	< Secondary education	412	155	873	1,440	7.54	**0.02**	130	34	309	473	8.07	0.02	282	121	564	967	2.66	0.26
	≥Secondary education	651	336	1,493	2,480			199	96	493	788			452	240	1,000	1,692		
Mother education	< Secondary education	562	237	1,261	2060	4.18	0.12	167	62	442	671	3.55	0.17	395	175	819	1,389	2.79	0.25
	≥Secondary education	501	254	1,105	1860			162	68	360	590			339	186	745	1,270		
Father occupation	Unskilled labor	501	219	1,100	1820	1.40	0.84	158	62	374	594	1.26	0.86	343	157	726	1,226	1.45	0.83
	Skilled labor	428	201	951	1,580			133	50	316	499			295	151	635	1,081		
	Business/service	134	71	315	520			38	18	112	168			96	53	203	352		
Mother occupation	Unskilled labor	326	156	728	1,210	19.13	**0.04**	102	45	250	397	13.09	**0.04**	224	111	478	813	9.57	0.15
	Skilled labor	134	58	278	470			39	11	96	146			95	47	182	324		
	Housewife	470	239	1,161	1870			141	65	389	595			329	174	772	1,275		
	Business/service	133	38	199	370			47	9	67	123			86	29	132	247		
Physical activity	No	578	179	803	1,560	130.46	**<0.001**	196	42	259	497	75.77	**<0.001**	382	137	544	1,063	62.74	**<0.001**
	Yes	485	312	1,563	2,360			133	88	543	764			352	224	1,020	1,596		
Screen time	1–2 h	183	127	1714	2024	1727.30	**<0.001**	60	23	388	471	570.78	**<0.001**	123	104	1,326	1,553	1336.84	**<0.001**
	2–4 h	679	196	172	1,047			198	35	38	271			481	161	134	776		
	>4 h	129	5	56	190			44	3	15	62			85	2	41	128		
	<1 h	72	163	424	659			27	69	361	457			45	94	63	202		

Bold values indicate statistically significant results (*p* < 0.05).

The Strength of associations between gender, diet, parental education, occupation, physical activity and screen time with overweight/obese and underweight status in a sample of 3,920 individuals, stratified into children (6–11 years, *n* = 1,261) and adolescents (12–19 years, *n* = 2,659). Females showed significantly higher odds of being overweight/obese (OR = 1.60, *p* < 0.001) compared to males in the total population, with stronger effects in adolescents (OR = 2.39, *p* < 0.001). A vegetarian diet increased the odds of overweight/obese (OR = 1.59, *p* < 0.001) particularly in adolescents. Maternal occupation, especially housewife status (OR = 0.61, *p* < 0.001), was consistently protective against overweight/obese status across groups, while unskilled and skilled labor also showed reduced odds in the total population and children. Parental education had limited impact, with lower father’s education linked to reduced odds of overweight/obese (OR = 0.78, *p* = 0.02) in children. Father’s occupation showed no significant associations, underscoring the stronger influence of maternal factors, gender, and diet on BMI outcomes. Lack of physical activity significantly increased the odds of overweight/obese status in the total population (OR = 2.32, *p* < 0.001) and adolescents (OR = 3.09, *p* < 0.001), but not in children (OR = 1.12, *p* = 0.29). Excessive screen time (2–4 h: OR = 23.24, *p* < 0.001; >4 h: OR = 13.56, *p* < 0.001) was strongly associated with overweight/obese status in the total population, with pronounced effects in adolescents (2–4 h: OR = 69.66, *p* < 0.001; >4 h: OR = 39.22, *p* < 0.001). Moderate screen time (1–2 h) was protective against both overweight/obese (OR = 0.62, *p* < 0.001) in the total population, though effects varied by age group. These findings highlight physical activity and excessive screen time as critical modifiable risk factors for overweight/obese status, particularly in adolescents (Table 3).

**Table 3:** Strength of association and its odds ratio of overweight/obese and underweight with gender, diet, and parental factors and risk factors for total data and by age groups (6–11 years & 12–19 years).

Demographic characteristics/risk factors		Total (*n* = 3,920)				6–11 years (*n* = 1,261)				12–19 years (*n* = 2,659)			
		Overweight/obese		Underweight		Overweight/obese		Underweight		Overweight/obese		Underweight	
		OR (95%CI)	*p*-value	OR (95%CI)	*p*-value	OR (95%CI)	*p*-value	OR (95%CI)	*p*-value	OR (95%CI)	*p*-value	OR (95%CI)	*p*-value
Gender	Female	1.60 (1.38–1.84)	**<0.001**	1.67 (1.37–2.03)	**<0.001**	0.79 (0.61–1.02)	0.08	0.75 (0.51–1.08)	0.12	2.26 (1.90–2.70)	**<0.001**	2.39 (1.89–3.02)	**<0.001**
	Male	1		1		1		1		1		1	
Religion	Muslim	0.38 (0.24–0.60)	**<0.001**	0.12 (0.06–0.20)	**<0.001**	0.39 (0.22–0.69)	**0.01**	0.12 (0.05–0.30)	**<0.001**	0.30 (0.13–0.67)	**0.04**	0.08 (0.04–0.19)	**<0.001**
	Buddhist	0.22 (0.12–0.42)	**<0.001**	0.53 (0.31–0.92)	**<0.001**	0.31 (0.15–0.64)	**0.02**	0.70 (0.31–1.55)	0.38	0.12 (0.04–0.35)	**<0.001**	0.33 (0.14–0.80)	**0.02**
	Hindu	0.40 (0.27–0.60)	**<0.001**	0.19 (0.12–0.28)	**<0.001**	0.55 (0.34–0.92)	**0.02**	0.44 (0.23–0.83)	**0.01**	0.19 (0.09–0.41)	**<0.001**	0.05 (0.02–0.11)	**<0.001**
	Other	1		1		1		1		1		1	
Diet	Veg	1.59 (1.35–1.86)	**<0.001**	0.71 (0.57–1.33)	0.32	0.86 (0.64–1.14)	0.29	0.92 (0.61–1.39)	0.69	2.18 (1.80–2.64)	**<0.001**	0.68 (0.50–1.94)	0.56
	Mixed	1		1		1		1		1		1	
Father education	< Secondary education	1.08 (0.93–1.26)	0.30	0.78 (0.64–0.97)	**0.02**	1.04 (0.80–1.36)	0.76	0.57 (0.37–0.87)	**0.007**	1.11 (0.92–1.32)	0.28	0.89 (0.71–1.13)	0.36
	≥Secondary education	1		1		1		1		1		1	
Mother education	< Secondary education	0.98 (0.85–1.13)	0.82	0.82 (0.67–0.99)	**0.04**	0.84 (0.64–1.09)	0.18	0.74 (0.51–1.07)	0.12	1.06 (0.89–1.26)	0.52	0.86 (0.68–1.07)	0.18
	≥Secondary education	1		1		1		1		1		1	
Father occupation	Unskilled labor	1.07 (0.85–1.34)	0.56	0.88 (0.65–1.18)	0.41	1.24 (0.82–1.88)	0.30	1.03 (0.57–1.81)	0.91	0.99 (0.78–1.31)	0.99	0.83 (0.59–1.17)	0.90
	Skilled labor	1.05 (0.84–1.33)	0.63	0.94 (0.70–1.27)	0.67	1.23 (0.81–1.88)	0.31	0.99 (0.75–1.78)	0.96	0.98 (0.74–1.30)	0.90	0.91 (0.64–1.29)	0.60
	Business/service	1		1		1		1		1		1	
Mother occupation	Unskilled labor	0.67 (0.52–0.87)	**0.02**	1.12 (0.76–1.65)	0.56	0.58 (0.37–0.90)	**0.02**	1.34 (0.62–2.88)	0.45	0.72 (0.52–0.98)	**0.04**	1.05 (0.67–1.66)	0.81
	Skilled labor	0.72 (0.53–0.98)	**0.03**	1.09 (0.70–1.71)	0.70	0.58 (0.34–0.98)	**0.04**	0.85 (0.34–2.17)	0.74	0.80 (0.55–1.15)	0.24	1.17 (0.70–1.97)	0.54
	Housewife	0.61 (0.47–0.77)	**<0.001**	1.08 (0.74–1.56)	0.69	0.52 (0.34–0.78)	**0.002**	1.24 (0.59–2.62)	0.57	0.65 (0.48–0.88)	**0.01**	1.02 (0.66–1.58)	0.91
	Business/service	1		1		1		1		1		1	
Physical activity	No	2.32 (2.00–2.70)	**<0.001**	1.12 (0.91–1.36)	0.29	3.09 (2.37–4.02)	**<0.001**	1.01 (0.68–1.48)	0.98	2.03 (1.70–2.43)	**<0.001**	1.15 (0.91–1.45)	0.26
	Yes	1		1		1		1		1		1	
Screen time	1–2 h	0.62 (0.47–0.84)	**<0.001**	0.19 (0.15–0.24)	**<0.001**	2.06 (1.28–3.32)	**<0.001**	0.31 (0.19–0.51)	**<0.001**	0.13 (0.08–0.20)	**<0.001**	0.05 (0.03–0.08)	**<0.001**
	2–4 h	23.24 (17.21–31.40)	**<0.001**	2.96 (2.26–3.90)	**<0.001**	69.66 (41.29–117.52)	**<0.001**	4.82 (2.85–8.16)	**<0.001**	5.02 (3.28–7.71)	**<0.001**	0.81 (0.54–1.20)	0.28
	>4 h	13.56 (9.08–20.25)	**<0.001**	0.23 (0.09–0.59)	**<0.001**	39.22 (19.38–79.33)	**<0.001**	1.04 (0.30–3.71)	0.95	2.90 (1.70–4.95)	**<0.001**	0.04 (0.02–0.14)	**<0.001**
	<1 h	1		1		1		1		1		1	

Bold values indicate statistically significant results (*p* < 0.05).

Adjusted odds ratios for overweight/obese status for screen time, by adjusted for gender, religion, diet, parental education, parental occupation, physical activity in the total population (*n* = 3,920) and age groups (6–11 years, *n* = 1,261; 12–19 years, *n* = 2,659). Excessive screen time significantly increased the odds of overweight/obese status in the total population (2–4 h: Adj. OR = 35.69, *p* < 0.001; >4 h: Adj. OR = 28.80, *p* < 0.001), with the strongest effects in adolescents (2–4 h: Adj. OR = 88.58, *p* < 0.001; >4 h: Adj. OR = 69.45, *p* < 0.001). Moderate screen time (1–2 h) was protective against underweight status across all groups (total: Adj. OR = 0.17, *p* < 0.001; adolescents: Adj. OR = 0.08, *p* < 0.001) but showed variable effects on overweight/obese status, increasing odds in adolescents (Adj. OR = 2.42, *p* < 0.001) while being protective in children (Adj. OR = 0.23, *p* < 0.001). These adjusted models emphasize the robust association between prolonged screen time and overweight/obese status, particularly in adolescents (Table 4).

**Table 4:** Adjusted odds ratios for overweight/obese and underweight status with screen time duration, adjusted for other risk factors, stratified by total population and age groups (6–11 and 12–19 years).

Exposure variable		Total (*n* = 3,920)				6–11 years (*n* = 1,261)				12–19 years (*n* = 2,659)			
		Overweight/obese		Underweight		Overweight/obese		Underweight		Overweight/obese		Underweight	
		Adj. OR (95%CI)	*p*-value	Adj. OR (95%CI)	*p*-value	Adj. OR (95%CI)	*p*-value	Adj. OR (95%CI)	*p*-value	Adj. OR (95%CI)	*p*-value	Adj. OR (95%CI)	*p*-value
Screen time	1–2 h	0.83 (0.61–1.13)	0.24	0.23 (0.18–0.30)	**<0.001**	2.42 (1.40–4.19)	**<0.001**	0.25 (0.14–0.43)	**<0.001**	0.17 (0.11–0.28)	**<0.001**	0.08 (0.05–0.11)	**<0.001**
	2–4 h	35.69 (25.35–50.25)	**<0.001**	4.01 (2.95–5.48)	**<0.001**	88.58 (47.35–165.68)	**<0.001**	4.40 (2.38–8.11)	**<0.001**	11.88 (7.23–19.51)	**<0.001**	1.52 (0.96–2.38)	0.28
	>4 h	28.80 (17.82–46.54)	**<0.001**	0.42 (0.16–1.09)	0.07	69.45 (28.27–170.62)	**<0.001**	1.47 (0.37–5.41)	0.61	9.22 (4.83–17.62)	**<0.001**	0.08 (0.02–0.32)	**<0.001**
	<1 h	1		1		1		1		1		1	

* Adjusted for gender, religion, diet, father education, mother education, father occupation, mother occupation, physical activity. Bold values indicate statistically significant results (*p* < 0.05). The asterisk (*) denotes statistically significant standardized path coefficients in the structural equation model (*p* < 0.05).

**Figure 1:**
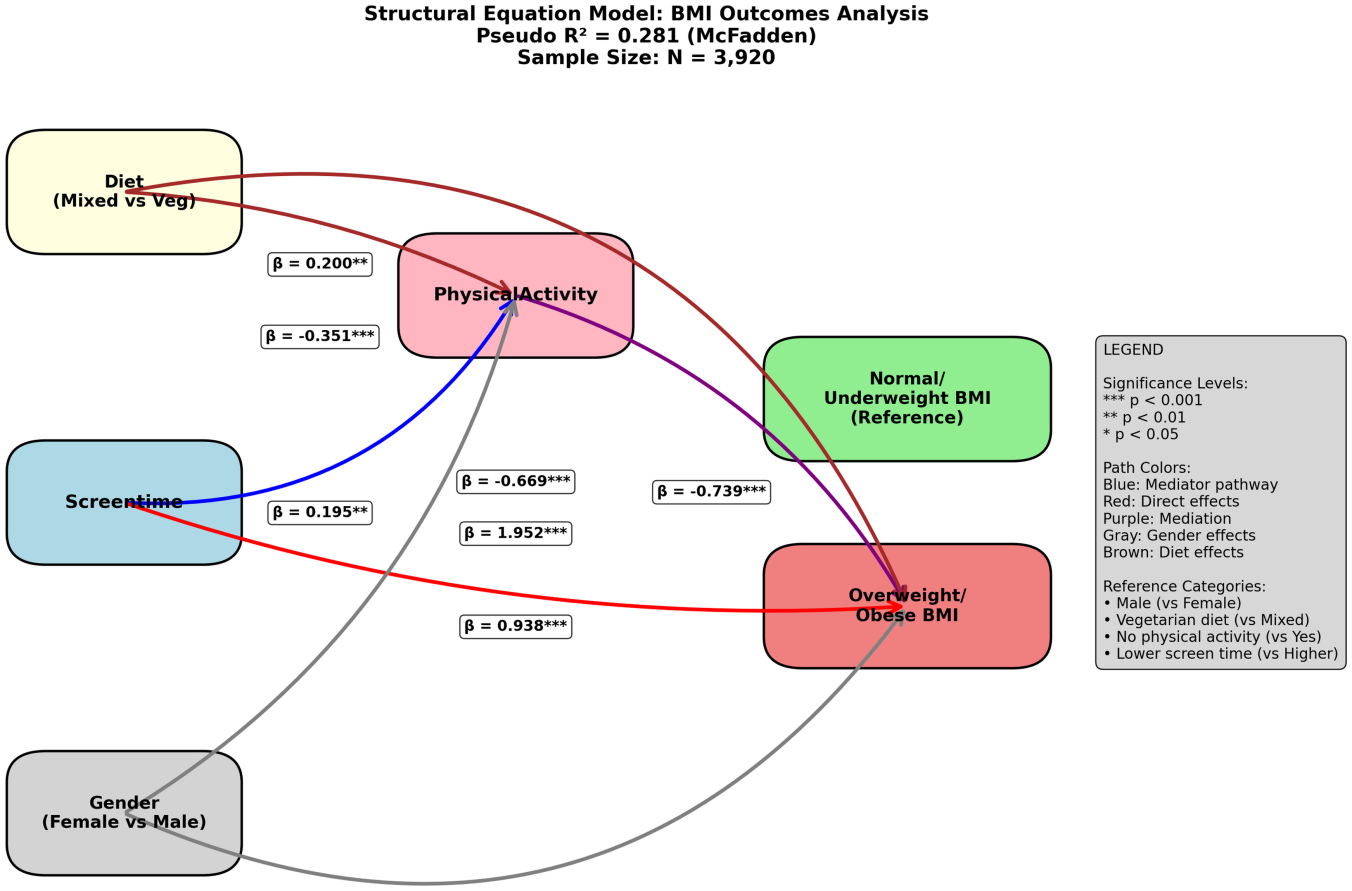
Structural equation model (SEM) of relationships between diet, screen time, gender, physical activity, and BMI Outcomes in the total population (*n* = 3,920).

The structural equation model (SEM) analysis, conducted with a sample size of 3,920, examines the relationships between diet (mixed vs. vegetarian), screen time, gender (female vs. male), physical activity, and BMI outcomes (normal/underweight vs. overweight/obese), explaining 28.1% of the variance in BMI (McFadden’s Pseudo R^2^). our study findings indicate that higher physical activity (*β* = −0.351, *p* < 0.001) and lower screen time (β = −0.669, *p* < 0.001) are significantly associated with normal/underweight BMI, while a vegetarian diet (β = 0.200, *p* < 0.001) and female gender (β = 1.952, *p* < 0.001) show positive direct effects on this outcome. Screen time and gender also mediate effects through physical activity (blue and purple paths), with significant direct effects of diet (β = 0.938, *p* < 0.001) and screen time (β = 0.195, *p* < 0.05) on BMI categories. These results suggest that promoting physical activity and reducing screen time, alongside dietary considerations, could be effective strategies for managing BMI, with gender playing a notable mediating role (Figure 1).

**Figure 2:**
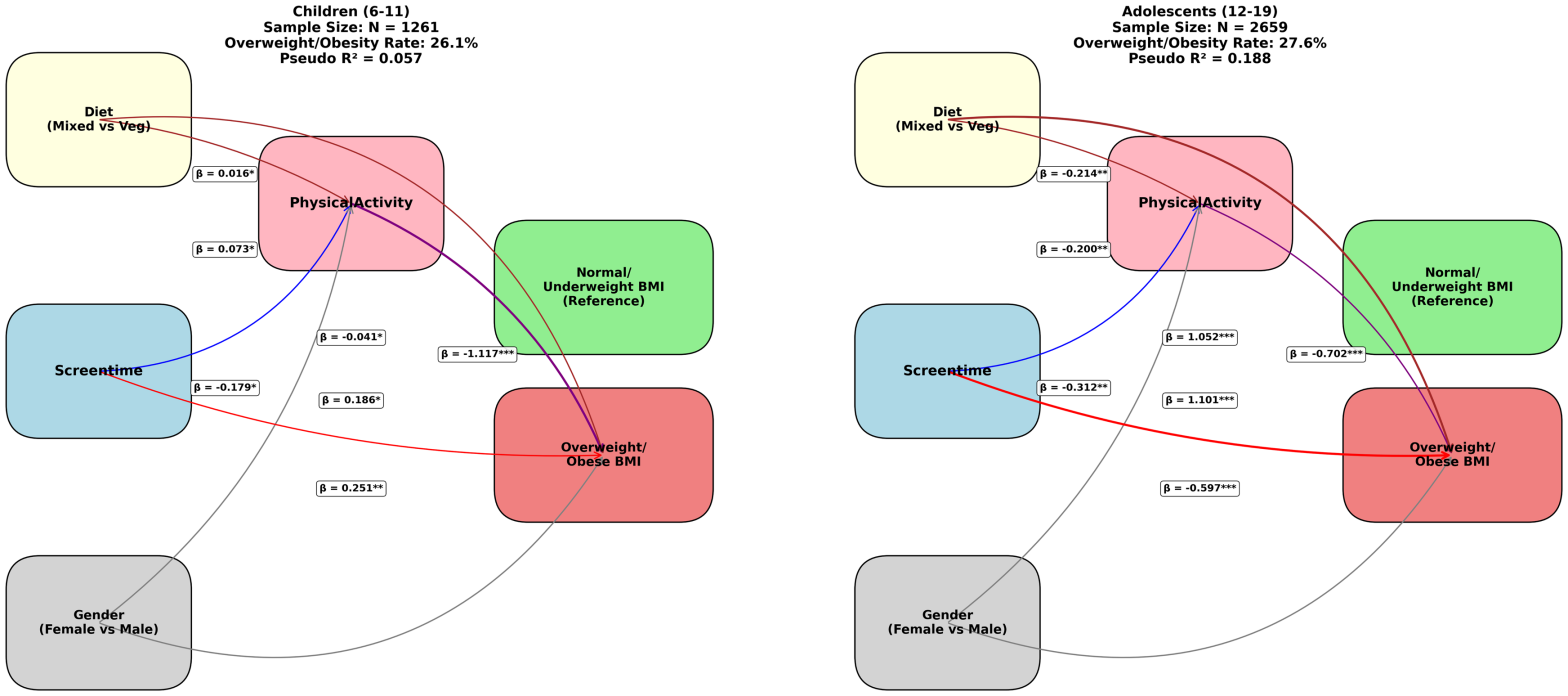
Structural equation model (SEM) comparing relationships between diet, screen time, gender, physical activity, and BMI outcomes across children (6–11 years, *n* = 1,261) and adolescents (12–19 Years, *n* = 2,659).

The structural equation model (SEM) analysis compares the relationships between diet (mixed vs. vegetarian), screen time, gender (female vs. male), physical activity, and BMI outcomes (normal/underweight vs. overweight/obese) across children (6–11 years, *N* = 1,261) and adolescents (12–19 years, *N* = 2,659), with Pseudo R^2^ values of 0.057 and 0.188, respectively. In children, higher physical activity (*β* = −0.016, *p* < 0.05) and lower screen time (β = −0.073, *p* < 0.001) are associated with normal/underweight BMI, while a vegetarian diet (β = 0.179, *p* < 0.001) and screen time (β = 0.186, *p* < 0.001) positively influence overweight/obese BMI. In adolescents, the effects are more pronounced, with physical activity (β = −0.200, *p* < 0.001) and screen time (β = −0.070, *p* < 0.001) showing stronger associations with normal/underweight BMI, and a vegetarian diet (β = 0.214, *p* < 0.001) and screen time (β = 1.101, *p* < 0.001) significantly linked to overweight/obese BMI. These findings highlight age-specific impacts, suggesting that interventions targeting physical activity and screen time reduction may be particularly effective in adolescents, with dietary influences varying across age groups (Figure 2).

## Discussion

The rising prevalence of overweight and obesity among children and adolescents has emerged as a significant public health concern, particularly in urban settings where lifestyle factors such as increased screen time and reduced physical activity are prevalent. This cross-sectional study, conducted among 3,920 students aged 6–19 years in urban schools of Pune, India, investigated the association between daily screen time and the risk of overweight and obesity, while also exploring the influence of socio-demographic factors such as age, gender, and socioeconomic status on screen time, physical activity, and obesity.

### Sociodemographic factors

In our study significant gender difference was observed in the prevalence of overweight and obesity among adolescents aged 12–19 years, with boys being considerably more likely to be overweight or obese than girls. This finding is consistent with results from a National survey conducted in China (6), which also reported higher rates of overweight and obesity among boys compared to girls. In contrast, Laurson et al. (7) found that obesity was more prevalent among girls than boys, a discrepancy that may be attributed to differences in sample size or population characteristics. Additionally, a study by Bai et al. (8) supported our findings, reporting higher obesity rates specifically in the 12–15-year age group.

Parental factors also play a role in children’s lifestyle habits and weight status. Our findings suggest that a mother’s occupation, specifically being a housewife, was associated with lower odds of childhood overweight/obesity (OR = 0.61). Several studies have examined the relationship between socioeconomic status (SES), parental education, and childhood overweight and obesity. In a study by Parikka et al. (9), both maternal and paternal education among younger boys showed significant direct and indirect associations with overweight. For older boys, paternal education remained directly and indirectly associated with overweight, whereas maternal education only showed an indirect inverse association. Similarly, a German cohort study by Ungehuelsing et al. (10) demonstrated that higher parental education levels and reduced screen time were associated with lower BMI among children and adolescents.

Evidence from Mumbai (11), India, revealed varying effects of SES indicators on obesity risk by gender. Among girls, higher maternal education was linked to lower odds of obesity. In contrast, among boys, higher family income and attendance at private schools, both indicators of higher SES, were associated with increased BMI and waist-to-height ratio (WHtR). This pattern suggests that in rapidly developing settings, rising affluence may contribute to higher obesity rates, potentially due to greater consumption of energy-dense foods and more sedentary lifestyles.

A national survey from China (12) observed that children and adolescents residing in urban areas, which typically have higher SES, had significantly greater odds of being overweight (OR = 1.15, *p* < 0.001) and obese (OR = 1.17, *p* < 0.001) compared to those in rural communities. Similarly, a study conducted in affluent private schools in Uttar Pradesh, India (13), reported higher rates of overweight (37.4%) and obesity (14.9%) than national averages for the region, suggesting a positive association between higher SES and obesity in that context.

Interestingly, the Mumbai (11) study further found that having a mother who was a housewife was associated with lower odds of overweight and obesity in children. Although the study did not elaborate on the reasons behind this association, it may reflect differences in family structure, household routines, or resource management.

In sum, across various studies and settings, SES—particularly parental education, occupation, and family income—emerges as an important determinant of overweight and obesity in children and adolescents. Meta-analyses and studies adjusting for these factors, including analyses of screen time and area-level disadvantage (such as the SEIFA Index in Australia), highlight the recognized influence of socioeconomic variables on obesity-related outcomes (14).

The relationship between screen time and age among adolescents is complex, with patterns varying by country, age group, and method of measurement.

In European countries, studies (15) involving children aged 6–9 years found that screen exposure starts early in life; however, no clear connection was seen between a child’s age and the amount of time spent on screens. Other factors, such as the mother’s age, influenced screen time—for example, children with older mothers (aged 45–54 years) were less likely to spend more than two hours per day on screens. Despite these insights, the average age of children studied was young (about 8 years), and the study concluded that age itself was not a significant factor for screen time within this age group.

In contrast, studies from India and China reveal more variation with age. In Mumbai (11), among adolescents aged 10–15, girls reported significantly more screen time than boys. Older adolescents (13–15 years) had higher screen time—as well as poorer sleep quality—than younger ones (10–12 years) (11). In Uttar Pradesh (13), screen time was widespread among school children aged 8–15 years, with the majority exceeding daily recommendations and an average of over 3 h each day. Age was found to significantly influence BMI, with screen time also playing an independent role. Although the study did not divide screen time results by narrower age groups, it suggested that the risk of obesity related to screen time seems to increase by age 12–13, emphasizing the need for preventive actions at even younger ages.

Studies from China (12), examining children and adolescents aged 7–19 years, found a mixed, non-linear link between age and screen time. For example, boys were less likely than girls to meet recommended limits on screen time. Adolescents in lower secondary school (junior middle school) were less likely to follow guidelines compared to primary school children, but in higher secondary school, there was some improvement. A 2016 study also noted that urban junior high school students were less likely to stay within screen time recommendations than their rural peers, suggesting that both age and living environment affect screen behaviors.

In the United States (16), average screen time among 10–14-year-olds was notably high (6.5 h per day). Higher categories of screen time were linked to greater body mass index (BMI) and increased risk of overweight and obesity, even after controlling for age.

A meta-analysis combining data from multiple adolescent studies (ages 10–19) found a stronger association between high screen time and overweight/obesity in those younger than 15. One explanation is that younger adolescents often have more unstructured free time—much of which is spent watching television or using other screens.

Taken together, these findings show that while high levels of screen time are common across different populations, the relationship between age and screen time is not straightforward. Some regions and age groups show clear trends of increasing screen time with age, while others do not. Additionally, younger adolescents may be more sensitive to the negative health effects of excessive screen use. Therefore, age is an important factor to consider when designing strategies to manage screen time and prevent obesity, and these approaches may need to be adjusted for different cultures and developmental stages.

### Physical activity and BMI

There is a consistent inverse relationship between physical activity and BMI in children and adolescents: higher levels of physical activity are generally linked to lower BMI and lower risk of overweight or obesity. However, the strength of this association and its interaction with age and screen time can differ across populations and studies.

In Mumbai (11), India, among adolescents aged 10–15 years, low physical activity (less than 60 min of moderate-to-vigorous activity per day) strongly predicted higher BMI and obesity in both girls (OR = 1.78) and boys (OR = 2.10). The risk was even higher when low physical activity was combined with high screen time. Additionally, older adolescents (13–15 years) were less active than younger ones (10–12 years), suggesting a decline in physical activity as children grow older.

In Uttar Pradesh (13), India, normal-weight children had much higher levels of physical activity than their overweight or obese peers. Most of the participants (97.7%) reported low physical activity, and nearly 69% of obese children were active for less than 60 min per day. Physical activity was shown to significantly and negatively affect BMI, meaning that more active children tended to have lower BMI.

In China (6), only about one-third of children and adolescents aged 7–19 years met the recommended levels of physical activity. Older students were much less likely to meet these guidelines compared to younger children, again showing that physical activity tends to decrease with age.

In Australia, although low physical activity was linked to a higher risk of overweight and obesity, the association was weaker than that of screen time. Physical activity and screen time were negatively related—in other words, children who spent more time on screens were generally less active. Notably, being physically active did not always prevent overweight or obesity, especially if screen time was also high.

In the United States (16), lower step counts (a measure of physical activity) were linked to higher BMI and greater risk of overweight and obesity. However, high physical activity did not fully protect against weight gain for adolescents who also had high screen time. Other American studies found that physical activity was important for cardiorespiratory fitness, but its impact on weight was reduced or even disappeared when screen time was taken into account.

When comparing all these findings, most studies agree that physical activity helps reduce the risk of overweight and obesity, but this effect can be weakened by high screen time. The decline in physical activity with age during adolescence is also a consistent trend. Overall, effective interventions to prevent childhood obesity should address both physical activity and screen time together, since being active alone may not be enough if children are also spending long hours on screens. This connection is often explained by several mechanisms:

1. Displacement of physical activity: screen time can replace time that would otherwise be spent on active play and reduce overall energy expenditure.
2. Increased snacking or unhealthy eating: adolescents often consume energy-dense foods while using screens, often in the absence of hunger.
3. Exposure to advertisements for unhealthy foods: screen time exposes children to advertisements for unhealthy foods, which can influence food choices. This indicates that the relationship between screen time and weight status involves mechanisms beyond merely reducing physical activity levels.

### Limitations

While our study offers valuable insights, it has several limitations that are common in similar research. The cross-sectional design restricts our ability to establish causality; we can observe associations but cannot definitively determine cause-and-effect relationships. Additionally, physical activity and screen time were self-reported, which may introduce inaccuracies due to recall bias, social desirability, or underreporting. Although BMI is a widely accepted measure, it reflects weight relative to height and does not differentiate between fat and muscle mass, presenting a known limitation. Future studies should incorporate objective measures of physical activity and screen time, use longitudinal designs to track behavior changes over time, and explore causal pathways more robustly. Including more specific factors—such as eating habits during screen use, distinctions between types of video games (active versus passive), and comprehensive assessments of sleep quality—could further deepen our understanding of the complex relationships involved.

## Conclusion and recommendation

Our findings highlight the urgent need for comprehensive public health strategies to address childhood overweight and obesity by simultaneously promoting reduced screen time and increased physical activity. Given the strong association between screen exposure and obesity risk—particularly when multiple unhealthy behaviors coexist—interventions must adopt a holistic, age-sensitive approach.

For children aged 6–11 years, strategies should emphasize the development of healthy eating habits early in life. Schools can play a pivotal role by encouraging students to bring nutritious, well-balanced tiffin meals from home and by refraining from operating canteens that sell calorie-dense or processed foods.

For adolescents aged 12–19 years, interventions should focus on enhancing physical activity through structured outdoor programs, sports participation, and active lifestyle promotion. When school canteens are present, enforcing strict healthy canteen policies and introducing regular “Fruit Day” initiatives can help normalize nutritious food choices. Additionally, recognizing and rewarding positive behaviors—such as through a “Healthy Champion” certificate for students who consistently follow healthy dietary and physical activity practices—can further motivate adolescents and foster a culture of wellness.

These age-appropriate, school- and family-based interventions, implemented with the active participation of parents as role models, can create supportive environments that reinforce healthy behaviors, balance energy intake and expenditure, and ultimately promote long-term well-being among children and adolescents.

## Data Availability

The original contributions presented in the study are included in the article/supplementary material, further inquiries can be directed to the corresponding author.
